# Measuring vitamin C in critically ill patients: clinical importance and practical difficulties—Is it time for a surrogate marker?

**DOI:** 10.1186/s13054-021-03670-x

**Published:** 2021-08-31

**Authors:** Sander Rozemeijer, Frans A. L. van der Horst, Angélique M. E. de Man

**Affiliations:** 1 Department of Intensive Care Medicine, Research VUmc Intensive Care (REVIVE), Amsterdam Cardiovascular Science (ACS), Amsterdam Infection and Immunity Institute (AI&II), Amsterdam Medical Data Science (AMDS), Amsterdam UMC, Location VUmc, Vrije Universiteit Amsterdam, Amsterdam, The Netherlands; 2 Department of Clinical Chemistry, Reinier Medical Diagnostic Center, Delft, The Netherlands

## Abstract

This article is one of ten reviews selected from the Annual Update in Intensive Care and Emergency Medicine 2021. Other selected articles can be found online at https://www.biomedcentral.com/collections/annualupdate2021. Further information about the Annual Update in Intensive Care and Emergency Medicine is available from https://link.springer.com/bookseries/8901.

## Introduction

Interest in intravenous vitamin C administration has rapidly increased in the field of critical care medicine over recent years. The first studies investigating the effect of intravenous vitamin C in septic (shock) patients showed a decrease in organ dysfunction, vasopressor dependency, and even a reduction in mortality [1–3]. Within a short period of time, multiple trials in septic patients were conducted to confirm these promising findings, but results were not uniform [4–12]. The inconsistencies in effects on outcome may partially be explained by differences in study design [8], in particular the dosing regimens (timing, duration and dose) and choice of co-medication. For example, vitamin C administration has been investigated alone, or in combination with thiamine and/or hydrocortisone, sometimes with uncontrolled use of hydrocortisone in the control group. There is also considerable variety among septic patients as sepsis is a heterogeneous syndrome. Therefore, some subgroups of patients might benefit more than others from intravenous vitamin C therapy. A recently published meta-analysis on mortality performed subgroup analyses and found a beneficial effect of vitamin C on short-term mortality (< 30 days). Additionally, survival was improved by a treatment duration of 3–4 days [13]. The results of vitamin C alone versus combination therapy were not different. A particular subgroup of interest is patients with vitamin C deficiency. None of the studies performed subgroup analyses on vitamin C deficient patients. This is unfortunate, but understandable, since the measurement of plasma vitamin C concentration is difficult.

In this chapter, we discuss the practical problems and pitfalls of measuring vitamin C and describe a novel potential surrogate marker that can estimate vitamin C status.

## Rationale of vitamin C administration

Vitamin C has pleiotropic functions in the human body, including anti-oxidative, anti-inflammatory and immune-supporting effects. It serves as a cofactor in the biosynthesis of norepinephrine and vasopressin, increases catecholamine sensitivity, protects the microcirculation, and improves wound healing [14]. Therefore, low plasma concentrations may have untoward effects in the ICU population.

Decreased plasma vitamin C concentrations are common in critically ill patients with sepsis, trauma, hemorrhage, post-cardiac arrest and burns [15–20]. In septic shock patients, hypovitaminosis C (< 23 μmol/l) and vitamin C deficiency (< 11 μmol/l) rates are as high as 88% and 38%, respectively [15]. Causes of deficiency are decreased intake and absorption, and, most importantly, increased metabolic consumption and reduced recycling due to overwhelming oxidative stress [14]. This increase in oxidative stress plays a key role in the pathophysiology of systemic inflammation and ischemia/reperfusion injury [17–20]. Vitamin C protects against oxidative injury to lipids, proteins and DNA by donating its electrons. When the amount of oxidative stress is overwhelming, vitamin C is readily consumed, recycling becomes insufficient and deficiency develops. As a result, the body’s protection against oxidative injury becomes inadequate.

This effect creates a strong rationale for vitamin C supplementation in vitamin C deficient patients with overwhelming oxidative stress [21]. Available literature has already addressed the importance of selecting vitamin C deficient patients by measuring baseline plasma vitamin C concentrations to create a clear difference in tissue and plasma vitamin C concentrations between control and treatment groups after supplementation [22]. In addition, measuring achieved plasma vitamin C concentrations could help to estimate optimal plasma concentrations. The direct radical scavenging effect of vitamin C increases with higher, supraphysiological concentrations [23]. Therefore, measuring vitamin C will provide more insight into the dose-concentration-clinical outcome relationship [22].

## Plasma vitamin C measurement

The determination of plasma vitamin C, or ascorbic acid, necessitiates considerable logistical and analytical effort.

### Drawing blood

**Fig. 1:**
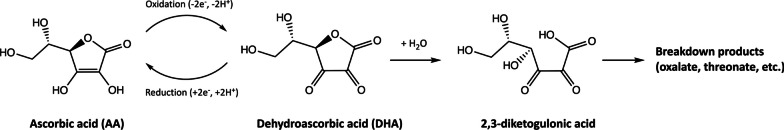
Degradation of vitamin C (ascorbic acid). By donating two electrons, dehydroascorbic acid (DHA) is formed. After hydrolysis, DHA is irreversibly degraded to 2,3-diketogulonic acid, leading to further breakdown products of vitamin C. The half-life of DHA is only minutes due to hydrolytic ring rupture. DHA can be reversibly reduced (recycling) by glutathione or enzyme-dependent mechanisms

To assess the *in vivo* vitamin C status, it is crucial to avoid *ex vivo* artefacts, i.e., the oxidation of vitamin C (ascorbic acid) to dehydroascorbic acid (DHA) and subsequent irreversible hydrolysis of DHA to 2,3-diketogulonic acid (Fig. 1). For this reason, samples have to be handled quickly after drawing blood to obtain a reliable vitamin C result. Several environmental factors have been shown to increase the rate of oxidation of vitamin C into DHA, of which the type of tube anticoagulant and surrounding temperature are the most prominent.

Vitamin C remains most stable in heparin during the first few hours [24, 25]. Ethylenediaminetetraacetic acid (EDTA) and serum whole blood are unstable at room temperature, with losses up to 15% and 20% within 2 h, respectively [24, 25]. In EDTA plasma, approximately 50% of vitamin C was lost at room temperature by 2 h, whereas in ice a significant decrease was observed from 4 h [25]. EDTA chelates of iron and copper are still redox active at physiological pH, and can still facilitate oxidation of ascorbate, particularly at room temperature. Therefore, samples should be handled quickly and kept cold during the whole period of handling and processing to reduce the loss of vitamin C after blood draw [25]. However, the use of ice water to reduce the temperature during transportation is difficult to manage. Nevertheless, although room temperature is a convenient logistical condition to transport samples, complying with the time constraints to process samples within less than 1 h to maintain sample integrity at room temperature is a considerable challenge for many hospitals.

### Sample treatment

The stability of vitamin C and DHA in plasma is significantly improved by acidification of the sample with, for example, metaphosphoric acid, trichloroacetic acid or perchloric acid, which also results in concomitant precipitation of the plasma proteins (deproteinizing). Acidification is commonly done after removal of the erythrocyte mass through centrifugation to ensure an efficient precipitation process of the remaining proteins. Deproteinization and stabilization can also be achieved by adding an organic modifier such as methanol, but these agents are less commonly used in the routine setting of hospital laboratories. The metal chelator EDTA or diethylene-triaminepentaacetic acid (DTPA) can be added at this point to further prevent *ex vivo* oxidation of vitamin C [25–27]. After acidification of the sample, this should be stored at low temperature, preferably at − 80 °C. Stability has been demonstrated for both vitamin C and DHA at − 80 °C for at least 5 years [28].

There is ongoing controversy in the literature about the generation of DHA in clinical samples [25]. Physiologically, the amount of DHA in plasma *in vivo* is < 2% of that of total vitamin C (ascorbic acid + DHA) [22]. Higher amounts of DHA can be expected in critically ill patients because of overwhelming oxidative stress with reduced recycling of DHA [14, 29], and in patients receiving vitamin C at pharmacological doses [22]. However, one study showed that the amount of DHA was low to negligible in clinical samples. Only samples that contained hemolysis had appreciable amounts of DHA, which was explained by the *ex vivo* release of iron from hemoglobin during the acidification step in the sample pretreatment [25]. An *in vitro* study showed that an increase in DHA due to ferric ions only occurred in plasma acidified with trichloroacetic acid or perchloric acid, not with metaphosphoric acid. Nevertheless, plasma hemoglobin catalyzed the oxidation of vitamin C in all acidic solutions [30]. Therefore, hemolysis should be avoided. Metaphosphoric acid is the best stabilizing agent of choice, as ferric ions, which are more easily released from transferrin in acidic solutions, did not accelerate the oxidation of vitamin C into DHA when using metaphosphoric acid [30]. One study showed a higher proportion of total vitamin C as DHA in metaphosphoric acid-acidified clinical samples compared to controls [31], implying that *in vivo* generation of DHA may also occur.

In a pharmacokinetic study we performed in critically ill patients, 10 patients received 2 g vitamin C and 10 patients received 10 g vitamin C intravenously, as a twice daily bolus or continuously over 48 h [32]. In the entire population, the median total vitamin C concentration at baseline was 22.7 [interquartile range (IQR) 14.7–39.5] μmol/l, and the median plasma DHA was 2.5 [0.9–5.1] μmol/l which was 10% (95% confidence interval (CI) 6.1–14.0%) of the total vitamin C (unpublished data). Patients receiving a bolus dosing regimen achieved peak plasma concentrations 1 and 2 h after infusion compared to the continuous dosing regimen in which peak plasma concentrations were achieved 24 and 48 h after infusion. In Table 1, total vitamin C (ascorbic acid + DHA), DHA, and DHA as a percentage of total vitamin C are shown at these peak moments. The absolute amount of DHA increased in patients receiving intravenous vitamin C therapy, but the percentage DHA of the total vitamin C remained comparable to baseline. Thus, DHA amounts greater than 2% may be caused by increased oxidative stress with reduced recycling due to critical illness, by supraphysiological plasma concentrations, and by the *ex vivo* oxidation of vitamin C despite adequate sample handling and processing [32].

**Table 1:** Total vitamin C and DHA in plasma samples of critically ill patients receiving vitamin C therapy. Data from [32]

Bolus dosing regimen	T = 1		T = 2	
	2 g (n = 5)	10 g (n = 5)	2 g (n = 5)	10 g (n = 5)
Total vitamin C, μmol/l	174 [163.8–254.8]	1101 [1067.5–1287.6]	136.1 [97.2–203.3]	733.1 [703.2–879.6]
DHA, μmol/l	12.7 [7.5–25.6]	100.3 [61.9–113.2]	8.7 [3.3–24.8]	63.6 [31.2–69.5]
**DHA, % of Tot (95% CI)**	**7.7 (2.8–12.7)**	**7.7 (4.1–11.2)**	**8.8 (0.8–16.8)**	**6.8 (3.6–10.0)**

Continuous dosing regimen	T = 24		T = 48	
	2 g (n = 5)	10 g (n = 5)	2 g (n = 5)	10 g (n = 5)
Total vitamin C, μmol/l	101.4 [15.8–134.9]	428.3 [352.8–612]	108.2 [42.0–174.1]	453.5 [318.9–1230.8]
DHA, μmol/l	11.7 [1.5–18.3]	31.9 [15.2–48.7]	8.4 [4.2–18.9]	18.6 [9.1–50.3]
**DHA, % of Tot (95% CI)**	**13.7 (5.3–22.0)**	**6.5 (3.1–9.9)**	**10.2 (2.5–17.9)**	**4.2 (0.1–8.3)**

Data are presented as median [IQR]

### Analysis

There are two distinct approaches to quantitative determination of vitamin C in plasma samples: enzymatic and chromatographic.

#### Enzymatic vitamin C assays

There are several commercially available kits based on enzymatic conversion of vitamin C resulting in a signal that can be detected photospectrometrically. Normally, the enzyme ascorbate oxidase is used in this type of assay. The common method for these assays is the enzyme-linked immunosorbent assay (ELISA), which is well suited for batchwise processing of samples, but less convenient for immediate determination of values in a few samples.

There have been several attempts to adapt these enzyme-based assays to auto-mated analyzers, but based on the methods reported in a European External Quality Assessment Scheme (Instand EQAS) enzyme-based assays are not routinely used within hospitals. If the clinical demand for immediate vitamin C determination increases, these enzyme-based assays could be used in point-of-care or centralized platforms because of their straightforward technical nature [33].

#### Chromatographic vitamin C assays

Quantitative ascorbic acid and DHA measurements are currently performed by high-performance liquid chromatography (HPLC) methods. HPLC methods are superior if multiple compounds with similar properties have to be analyzed or if there are many substances that might interfere with the quantification of a compound of interest.

After injection of the acidified sample into the HPLC instrument, the compounds are separated by passing through a column that differentially retains compounds based on their physical properties. As a result, at the end of the separation column, ascorbic acid and DHA can be detected selectively without interference of other compounds. Currently, there are two methods to detect ascorbic acid and DHA after passing through the column [34]. First, electrochemical detection, which uses the redox-properties of ascorbic acid and DHA, and second, ultraviolet (UV) detection, which is based on the UV absorption of these compounds. Despite the fact that both detection methods give identical results, UV detection is more widely used in the routine setting because of its relative technical simplicity [34]. Other detection techniques have been used, such as fluorescence detection after pre-column chemical modification of ascorbic acid and DHA into a fluorescent compound, but are less common. Colorimetric/fluorometric methods may generate higher DHA concentrations due to the lack of specificity of the method [25].

#### Chromatographical assessment of ascorbic acid, DHA and total vitamin C

To assess the total vitamin C status, both ascorbic acid and DHA have to be determined. Although in principle these compounds can be quantified simultaneously in a single HPLC run, this approach is not often used because there is no traditional need for the separate quantification of DHA to assess vitamin C deficiency in patients. Another reason is that it is technically much easier to determine only ascorbic acid instead of both ascorbic acid and DHA simultaneously. Therefore, medical laboratories have optimized their HPLC assays to optimally detect ascorbic acid and not the combination of ascorbic acid and DHA. A convenient way to solve this is to convert DHA into ascorbic acid in the sample prior to HPLC analysis. Any generated DHA that has not yet been hydrolyzed to the irreversible 2,3-diketogulonic acid can be reduced to ascorbic acid by adding a reducing agent, such as tris(2-carboxyethyl) phosphine hydrochloride (TCEP) or dithiothreitol (DTT). It is even possible to use pre-modified heparinized tubes with added DTT to immediately reduce any formed DHA [35]. In this manner, the total vitamin C concentration (ascorbic acid + DHA) in the sample can be measured. It has been shown that compared to its instability at room temperature, DHA is stable for several hours at 4 °C, for at least a year when stored non-acidified at − 80 °C [25], and for at least 5 years when stored acidified at − 80 °C [28]. Thus, if samples are appropriately handled and processed, it should be possible to recover any oxidized ascorbic acid with agents such as TCEP and DTT.

With respect to clinical utilization of the total vitamin C determination, response times have to be considered. If sample pre-treatment is limited to centrifugation and deproteination prior to analysis, this will take approximately 45 min, whereas the analytical procedure itself, including calculations and verification, will take an additional 30 min. So, under optimized and dedicated conditions, it will take over 1 h to obtain quantitative results.

Because of the analytical complexity, many clinical chemistry laboratories outsource the analysis of vitamin C to reference laboratories, making vitamin C determination unavailable for routine care. If they do supply this diagnostic service themselves, it is unlikely this it is available for immediate determinations. Moreover, the sample pre-treatment to safeguard correct determination of vitamin C is rather cumbersome and not easily accommodated into routine hospital logistics, especially not in intensive care and emergency departments. A point-of-care vitamin C measurement could therefore be useful, but such a measure is not available yet. A potential surrogate marker is the static oxidation-reduction potential (sORP), which can be measured quickly at the bedside.

## Static oxidation-reduction potential

**Fig. 2:**
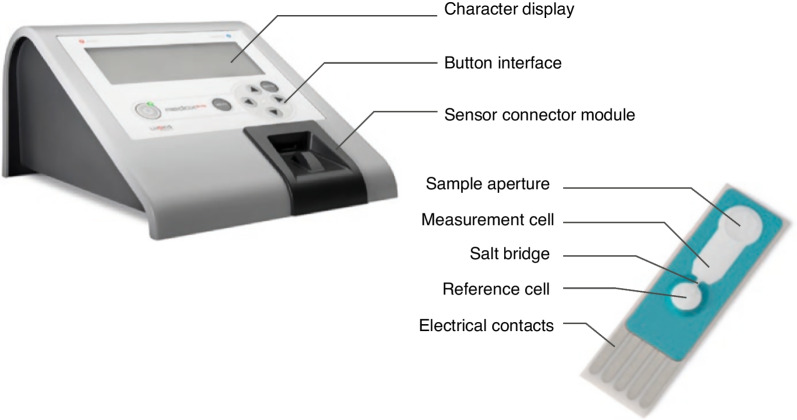
Image of the portable RedoxSYS analyzer and disposable strip. From [36] with permission

The sORP is a marker that reflects the overall amount of oxidative stress in the blood, as measured by the RedoxSYS Diagnostic System (Aytu Bioscience, Englewood, CO, USA) [36]. It consists of a portable RedoxSYS analyzer and a disposable sensor strip (Fig. 2). This point-of-care device quickly measures the net balance between the total amount of known and unknown oxidants and reductants in a biological sample, e.g., whole blood, plasma, serum or urine. The sORP results are shown on a small display screen within 4 min after applying approximately 30 μl of the sample to the sensor. The total time between obtaining blood and getting a sORP test result is less than 20 min. More detailed technical information about the test is available elsewhere [36, 37]. One of the advantages of measuring sORP is that it does not rely on a single biomarker of oxidative stress, such as lipid peroxidation. It provides a complete picture of the total amount of oxidative stress in the sample.

**Fig. 3:**
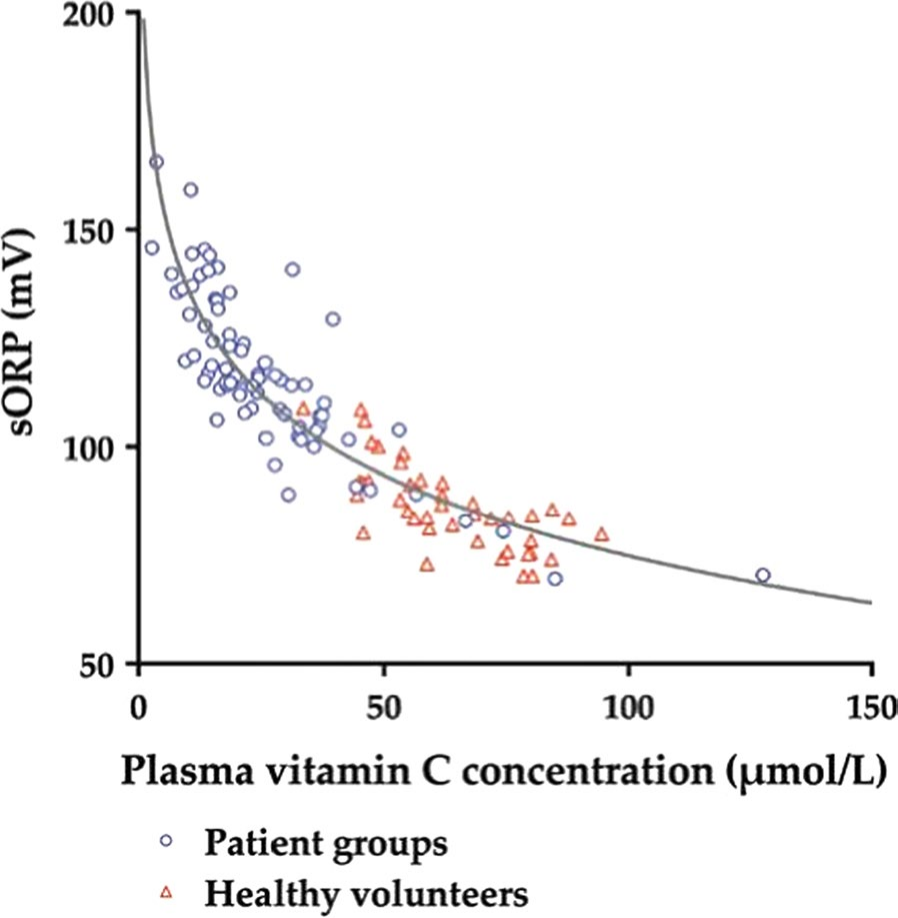
Scatter plot showing the association between the static oxidation-reduction potential (sORP) and plasma vitamin C concentration. From [17] with permission

sORP was strongly inversely associated with plasma vitamin C concentration in healthy volunteers and critically ill patients (Fig. 3) [17]. While sORP increased, plasma vitamin C concentration and total plasma antioxidant capacity decreased during the ICU stay. In patients who received vitamin C therapy, sORP decreased significantly. Furthermore, in previous *in vitro* studies, sORP also decreased after adding vitamin C [36–39]. This strong relationship is expected, because vitamin C is an excellent reducing agent [40]. Vitamin C is able to rapidly scavenge free radicals, and can be recycled afterwards. As a result, the total amount of oxidative stress will have a significant impact on the total amount of vitamin C and *vice versa*.

These results show that sORP has the potential for use to estimate plasma vitamin C concentrations and to screen for low vitamin C status. Preliminary data from our diagnostic accuracy study show that sORP estimates low plasma vitamin C concentrations accurately, whereas it is less precise at higher ranges. In contrast to the laborious handling, processing and analysis of samples to measure vitamin C, sORP is measured in non-acidified, non-reduced plasma within 20 min, directly after centrifugation and even after storage at − 80 °C for many years [17]. When used at the bedside, it is recommended that the sORP measurement is performed as soon as possible after drawing blood and centrifugation to minimize the *ex vivo* oxidation of vitamin C. Notwithstanding the short handling time, the sORP measurement can become very useful in intensive care and emergency medicine.

## Conclusion

This chapter underlines the potential clinical relevance of measuring plasma vitamin C concentrations in critically ill patients and the practical difficulties that go along with the currently available measurement. Multiple clinical studies have investigated the effects of intravenous vitamin C in critically ill patients, but results are not uniform. A possible explanation is the heterogeneity in study designs and included patients. Patients with vitamin C deficiency might benefit more from vitamin C therapy compared to non-deficient patients. Rapid plasma vitamin C measurement could identify this subgroup, but the ready oxidation of vitamin C *ex vivo* leads to several practical difficulties. Vitamin C measurement is therefore cumbersome, time consuming and not available for routine care. Proper blood handling, processing and analysis to estimate plasma vitamin C concentrations are crucial to obtain reliable results. It is recommended to use heparin-anticoagulated tubes, to process the samples within less than 1 h at low temperature, and to stabilize the sample through acidification and deproteinization with metaphosphoric acid. Oxidized vitamin C (DHA) can be recovered using a reducing agent such as DTT. The sORP can estimate vitamin C status at the bedside within 20 min using fresh unprocessed plasma samples. As this measurement is much more practical, especially for emergency medicine, sORP can serve as a surrogate marker for vitamin C allowing evaluation of the effectiveness of vitamin C therapy in the subgroup of patients with low vitamin C status.

## Data Availability

The data analysed during the current study are available from the corresponding author on reasonable request.
